# Adaptive and aberrant reward prediction signals in the human brain

**DOI:** 10.1016/j.neuroimage.2009.11.075

**Published:** 2010-04-01

**Authors:** Jonathan P. Roiser, Klaas E. Stephan, Hanneke E.M. den Ouden, Karl J. Friston, Eileen M. Joyce

**Affiliations:** a Institute of Cognitive Neuroscience, University College London, 17 Queen Square, London, WC1N 3AR, UK; b Wellcome Trust Centre for Neuroimaging, Institute of Neurology, University College London, 12 Queen Square, London, WC1N 3BG, UK; c Laboratory for Social and Neural Systems Research, Institute for Empirical Research in Economics, University of Zurich, Switzerland

**Keywords:** Adaptive reward learning, Aberrant reward learning, Dopamine, Striatum, Salience attribution test (SAT), Dorsolateral prefrontal cortex, Middle temporal gyrus

## Abstract

Theories of the positive symptoms of schizophrenia hypothesize a role for aberrant reinforcement signaling driven by dysregulated dopamine transmission. Recently, we provided evidence of aberrant reward learning in symptomatic, but not asymptomatic patients with schizophrenia, using a novel paradigm, the Salience Attribution Test (SAT). The SAT is a probabilistic reward learning game that employs cues that vary across task-relevant and task-irrelevant dimensions; it provides behavioral indices of adaptive and aberrant reward learning. As an initial step prior to future clinical studies, here we used functional magnetic resonance imaging to examine the neural basis of adaptive and aberrant reward learning during the SAT in healthy volunteers. As expected, cues associated with high relative to low reward probabilities elicited robust hemodynamic responses in a network of structures previously implicated in motivational salience; the midbrain, in the vicinity of the ventral tegmental area, and regions targeted by its dopaminergic projections, i.e. medial dorsal thalamus, ventral striatum and prefrontal cortex (PFC). Responses in the medial dorsal thalamus and polar PFC were strongly correlated with the degree of adaptive reward learning across participants. Finally, and most importantly, differential dorsolateral PFC and middle temporal gyrus (MTG) responses to cues with *identical reward probabilities* were very strongly correlated with the degree of aberrant reward learning. Participants who showed greater aberrant learning exhibited greater dorsolateral PFC responses, and reduced MTG responses, to cues erroneously inferred to be less strongly associated with reward. The results are discussed in terms of their implications for different theories of associative learning.

## Introduction

It is well established that mesolimbic dopamine transmission mediates the processes by which: (i) reinforcement learning occurs (Schultz et al., 1997; Tsai et al., 2009) and (ii) conditioned stimuli come to drive goal-directed behavior (Berridge and Robinson, 1998). A number of theorists have suggested that the spurious learning of contingencies between events that in fact co-occur only coincidentally might be related to dysregulated dopamine transmission (King et al., 1984; Gray et al., 1991; Shaner, 1999). This process has been hypothesized to contribute to the development of abnormal beliefs in psychotic disorders such as schizophrenia (see Corlett et al., 2007 for a review), which is associated with both dopaminergic abnormalities (Abi-Dargham, 2004) and reinforcement learning deficits (Waltz et al., 2007; Waltz and Gold, 2007). Integrating such findings with phenomenological accounts of psychosis, Kapur (2003) proposed the ‘aberrant salience’ hypothesis of psychosis, which explicitly links the aberrant signaling of motivational salience by dysregulated dopamine transmission to psychotic symptoms.

We recently provided the first evidence directly supporting the aberrant salience hypothesis using a novel behavioral paradigm, the Salience Attribution Test (SAT; Roiser et al., 2009). The SAT is a probabilistic reward learning task featuring compound cue stimuli that vary along two dimensions, one task-relevant and one task-irrelevant. ‘Adaptive’ reward learning refers to differences in ratings (the explicit measure of learning) and reaction times (the implicit measure of learning) along the task-relevant cue dimension, i.e. for high-probability reward cue features relative to low-probability reward cue features. ‘Aberrant’ reward learning is defined similarly, but along the task-irrelevant dimension, i.e. differences in ratings or reaction times between cue features that are both associated with 50% probability of reward. In our previous behavioral study, we found that schizophrenia patients with delusions scored significantly higher than those without delusions on our measures of aberrant learning.

Prior to future neurophysiological studies of patients with psychotic symptoms employing the SAT, we wished to investigate the neural mechanisms mediating adaptive and aberrant reward learning in healthy volunteers, Using functional magnetic resonance imaging (fMRI) predicted that (i) *adaptive* reward prediction signals (i.e. neural responses to high-probability reward cue features relative to low-probability reward cue features) would be reflected in hemodynamic responses in cortico–striatal–thalamic circuitry innervated by dopamine and previously implicated in reward processing; in particular the medial dorsal (MD) thalamus, ventral striatum and prefrontal cortex (PFC) (Brown et al., 1979; Alexander et al., 1986; Voorn et al., 1986; Oades and Halliday, 1987; Schultz et al., 1997; Knutson et al., 2004; Sanchez-Gonzalez et al., 2005; Garcia-Cabezas et al., 2007). We further predicted that (ii) *aberrant* reward prediction signals (i.e. differential neural responses to two different categories of cue features associated with identical reward probabilities) would be reflected in hemodynamic responses in separate but partially overlapping circuits, particularly in the dorsolateral PFC (DLPFC), which is hypothesized to play a central role in the optimization of stimulus–reward associations and resulting behavior (Montague et al., 2004; Grace et al., 2007).

## Materials and methods

### Participants

Twenty-three right-handed healthy volunteers, nineteen of whom were included in the final analysis (average age 27 years (SD 6.5 years); average IQ 102 (SD 8.8)) were recruited by advertisement. Exclusion criteria were: known psychiatric or neurological disorder; medical disorder likely to lead to cognitive impairment; IQ < 70; recent illicit substance use and first-degree relatives diagnosed with a psychotic illness. The absence of Axis-I psychopathology and alcohol- or substance-abuse/dependence was confirmed with the Mini International Neuropsychiatric Inventory (MINI: Sheehan et al., 1998). IQ was estimated using four sub-tests of the Wechsler Adult Intelligence Scale-Revised (Blyler et al., 2000).

Ethical approval was obtained from the Ealing & West London Mental Health Trust and National Hospital for Neurology and Neurosurgery & Institute of Neurology Research Ethics Committees. All participants provided written informed consent, were compensated £40 for their participation and could win up to another £20 on the SAT.

### Salience Attribution Test

#### Task structure

**Fig. 1:**
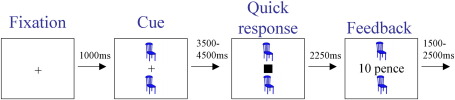
Salience Attribution Test. Participants were required to respond to the square as quickly as possible. On 50% of trials, participants won more money for quicker responses, with the probability of reward signaled by the cue appearing immediately prior to the probe.

The SAT, which included a standardized tutorial performed outside the scanner to familiarize participants with the game, has been described previously (Roiser et al., 2009; Fig. 1). On each trial, participants had to make a speeded response to the onset of a probe (a white square) in order to earn money, with more money earned for quicker responses. Prior to the main game, participants performed a practice session in the scanner, with no rewards and no cues, on which they were simply required to respond as quickly as possible to the onset of the probe. From this practice session each participant's mean reaction time (RT) and standard deviation of the ten fastest trials (SDF) was calculated in order to calibrate task difficulty on a participant-by-participant basis (see below) (Fig. 1; Roiser et al., 2009).

On the main game, monetary reward was available on 50% of trials, and on these trials participants won between 5 and 100 pence depending on the speed of their response, with feedback provided at the end of each trial. On rewarded trials where participants either made no response or responded after the probe had disappeared, the message “Missed: 5 pence” was displayed. If participants responded prematurely (< 100ms after the onset of the probe), the message displayed was “Too early: 5 pence”. On rewarded trials where participants responded before the probe disappeared, but slower than their mean RT, the message “Hit—good: 10 pence” was displayed. When participants responded more quickly than their mean RT, the message “Quick—very good: X pence” was displayed (for responses up to 1.5 SDFs faster than their mean RT) and “Very quick—excellent: X pence” (for responses faster than their mean RT by at least 1.5 SDFs). The reward was scaled according to *X* = 10 + 90 × (practice mean RT − trial RT) / (3 × SDF), up to a maximum of 100 pence. For example, a response 1 SDF faster than the mean was rewarded with 40 pence, a response 2 SDFs faster was rewarded with 70 pence, and any responses 3 SDFs or faster than the mean were rewarded with 100 pence. On the 50% of trials that were not rewarded, the message “Sorry—no money available” was displayed, regardless of the speed of response.

The likelihood of reward on a given trial was signaled by one of four categories of cues that appeared on-screen before the onset of the probe. The cues varied on two different visual dimensions: color (blue or red) and shape (animal or household object). Therefore, there were four different types of cue: blue animals; red animals; blue household objects and red household objects. Each cue set consisted of 16 different pictures, each of which was presented once per block. One of the cue dimensions (e.g. ‘color’) was task-relevant, so that one level of the dimension was rewarded on 28/32 of the trials (e.g. reward on 14/16 ‘blue animal’ and 14/16 ‘blue household object’ trials, corresponding to 87.5% of all ‘blue’ trials), while only 4/32 trials of the other level were rewarded (e.g. red on 2/16 ‘red animal’ and 2/16 ‘red household object’ trials, corresponding to 12.5% of all ‘red’ trials). The other dimension (in this example ‘shape’) was task-irrelevant, so that reward occurred on 16/32 trials of both levels of the dimension (in this example corresponding to 50% of all ‘animal’ and 50% of all ‘household object’ trials). Participants were not informed of these contingencies. Three blocks of 64 trials were performed, with identical reward contingencies on each block of the game. The task-relevant and task-irrelevant dimensions were counterbalanced across subjects (see below).

#### Trial structure

At the beginning of each trial a fixation-cross appeared. After 1 s, while the fixation-cross remained on-screen, one of the four cues was displayed at the left and right of the screen and remained on-screen until the end of the trial. After a period of time that varied randomly across trials (between 3.5 and 4.5 s) the probe appeared, replacing the fixation-cross, and participants attempted to respond before it disappeared using the index finger of their right hand on an MRI compatible button-box. The onset of the probe was therefore unpredictable, ensuring that participants were unable to anticipate its appearance. The duration of the probe also varied randomly across trials, and was calibrated for each participant separately from their own practice session data, with limits 2 SDFs either side of the practice mean RT. After 2.25 s feedback was presented for 1.5 s as described above. On rewarded trials, a tone with frequency proportional to the amount of money won on that trial sounded at feedback. After feedback, a blank screen of variable duration was inserted to result in a constant inter-trial interval of 9.25 s (Fig. 1).

Four different versions of the SAT were used, counterbalanced across participants, each with a different stimulus feature (blue, red, animal or household object) rewarded with high probability. Each participant was administered the same version for each block of the SAT. At the end of each block, participants indicated, using 100 mm visual analogue scales (VAS), their estimate of the reward probabilities for each of the four different cues, ranging from 0% (never associated with money) to 100% (always associated with money), such that 1 mm corresponded to 1%.

#### Outcome variables

Measures of reward learning were calculated for each block according to VAS ratings and RTs. *Adaptive reward learning* was defined as the increase in probability rating (the explicit measure of adaptive reward learning), or speeding of responses (the implicit measure of adaptive reward learning), for high-probability reward trials relative to low-probability reward trials (e.g. ‘blue’ relative to ‘red’ in the above example). *Aberrant reward learning* was defined as the *absolute* difference in VAS rating (the explicit measure of aberrant reward learning) or RT (the implicit measure of aberrant reward learning) between the two levels of the task-irrelevant stimulus dimension (e.g. the unsigned difference between ‘animal’ and ‘household object’ in the above example). The number of premature responses and omissions was also recorded for each stimulus type on each block.

### MRI image acquisition

Blood-oxygen-level-dependent (BOLD) responses were measured while participants performed the SAT using a 3 T head scanner (Magnetom Allegra, Siemens Medical, Erlangen, Germany) operated with its standard head transmit-receive coil. We acquired gradient-echo T2⁎-weighted echo-planar images (EPI) utilizing a single-shot gradient-echo sequence optimized to reduce signal dropout in the orbitofrontal cortex (Weiskopf et al., 2006). Forty-two oblique transverse slices of 2 mm thickness and a 1 mm gap between slices were acquired with 3 mm in-plane resolution; repetition time (TR) = 2.73 s; echo time (TE) = 30 ms; bandwidth (BW) = 3551 Hz/pixel; bandwidth in phase encoding direction (BWPE) = 47.3 Hz/pixel; phase encoding direction = anterior–posterior; field of view (FOV) = 192 × 192 mm^2^; matrix size 64 × 64 with fat suppression. BOLD sensitivity losses in the orbitofrontal cortex due to susceptibility artifacts were minimized by applying a z-shim gradient moment of −2 mT/m lasting 1 ms, with a slice tilt of −30° to the anterior commissure–posterior commissure line and a positive PE gradient polarity (Weiskopf et al., 2006). EPI magnitude images were reconstructed from the complex k-space raw data using a generalized reconstruction method based on the measured EPI k-space trajectory to minimize ghosting. EPI data acquisition was monitored online using a real-time reconstruction and quality assurance system (Weiskopf et al., 2007). The first five images in each series were discarded to allow for T1 saturation. T1-weighted structural scans were also acquired for each participant, with resolution 1 mm^3^; TR = 7.92 ms; TE = 2.4 ms; TI = 910 ms; BW = 195 Hz/Px; *α* = 15°.

### fMRI analysis

EPI data were analyzed using an event-related design with Statistical Parametric Mapping (SPM5—www.fil.ion.ucl.ac.uk/spm). Briefly, after discarding the first five images of each session to allow for T1 saturation, the remaining images were unwarped and realigned to the sixth image in the series, spatially normalized to the Montreal Neurological Institute (MNI) template and smoothed with a Gaussian kernel at 8 mm full half-width maximum (FWHM). Maximum likelihood parameter estimates were calculated at each voxel using the general linear model and an AR(1) model of serial correlations. In this model, four ‘cue’ regressors, representing the different cue types, an ‘outcome’ regressor representing the outcome of each trial and its parametric modulation according to reinforcement magnitude (0–100 pence), were created by convolving the onset of each event (duration 2 s for cue regressors, 1.5 s for outcome regressors) with a set of temporal basis functions (see below). Cues on which participants failed to respond entirely were excluded from the analysis, due to the possibility that participants were not attending during the trial. However, the outcomes on these trials were modeled, since the pitch of the tone played during feedback would alert participants to the magnitude of the reward, even if their eyes were closed. Trials on which participants responded extremely prematurely (< 1250ms) were also excluded, so as to avoid contaminating the measurement of cue-associated responses with movement-associated responses, though outcomes on these trials were modeled. Reaction times were modeled implicitly via parametric modulation by reinforcement magnitude (see above); since reward magnitude was dependent on reaction time, an additional regressor of reaction times would have resulted in very highly correlated regressors, drastically reducing statistical efficiency. To account for inter-regional and inter-subject variability in the shape of the hemodynamic response function (HRF), we used a set of temporal basis functions that included a canonical HRF as well as its temporal and dispersion derivatives. The model additionally included drift terms up to 1/128 Hz to remove low-frequency components, and global confounds were removed using global normalization.

At the first (within-subject) level, three contrast images were generated per participant, representing:

• Parametrically modulated reward delivery
• Adaptive reward prediction responses (high-probability reward cue features minus low-probability reward cue features across the task-relevant dimension—e.g. ‘blue’ cues minus ‘red’ cues)
• Aberrant reward prediction responses (subjective ‘high-probability’ reward cue features minus subjective ‘low-probability’ reward cue features across the task-irrelevant dimension—e.g. ‘animal’ cues minus ‘household object’ cues)

To calculate the aberrant reward prediction contrast images, subjective ‘high-probability’ and ‘low-probability’ cue types were defined for each block separately on the basis of each participant's VAS ratings (i.e. the direction of the explicit measure of aberrant reward learning). So if in the above example a subject had rated the animal cues as having a higher reward probability than household object cues in a particular block, all animal stimuli were defined as ‘high’ probability for that block in the aberrant reward prediction contrast.

Following first-level analysis, the contrast images of each participant were checked manually to ensure accurate normalization to the MNI template, and to exclude participants with corrupted images or artifacts resulting from excessive head-movement. This led to the exclusion of 4 participants from the group-level analyses. The contrast images from the remaining 19 participants were subjected to analysis at the group level using the summary statistics approach to random-effects analyses.

Three random-effects group-level analyses were performed, each entailing a one-sample *t*-test on the contrast images from the contrasts listed above. To examine the effects of inter-individual differences in reward learning we included the explicit measures of adaptive and aberrant reward learning, assessed by VAS ratings and averaged over all three blocks, as explanatory variables in the (second and third) *t*-tests of the corresponding hemodynamic responses. These maps were thresholded at an uncorrected level of *p* = 0.001.

We were particularly interested in exploring responses in the ventral striatum and MD thalamus, since these regions have previously been implicated in reward processing (Knutson et al., 2004; Pessiglione et al., 2006) and are innervated by dopamine (Brown et al., 1979; Knutson et al., 2004; Sanchez-Gonzalez et al., 2005; Garcia-Cabezas et al., 2007). For analysis of responses in the ventral striatum for the adaptive and aberrant reward prediction contrasts, we defined spheres of radius 8 mm as volumes of interest (VOIs) centered on the peak voxels identified by the orthogonal analysis of parametric modulation of reward outcome (right: [*x* = 15; *y* = 18; *z* = − 6]; left: [*x* = − 9; *y* = 12; *z* = − 3]). For analysis of responses in the MD thalamus for the covariate analyses, we defined a sphere of radius 8 mm as a VOI centered on the peak voxel identified in the analysis of adaptive reward prediction (right: [*x* = 3; *y* = − 9; *z* = 9]; left: [*x* = − 3; *y* = − 9; *z* = 9]). Note that in both cases the contrast used to define the VOI was orthogonal to the contrast of interest.

We discuss all effects surviving either voxel-level or cluster-level family-wise error (FWE) correction for whole-brain multiple comparisons (WBC), or small-volume corrected (SVC) voxel-level FWE correction for multiple comparisons across the VOIs defined above. For completeness we list all clusters comprising 10 contiguous voxels at a *t*-threshold of *p* < 0.001 (uncorrected) in Tables S1–S3. Anatomical localization was performed with reference to the atlas of Mai et al (2003), after transforming MNI coordinates to the stereotaxic space of Talairach and Tournoux (http://imaging.mrc-cbu.cam.ac.uk/imaging/MniTalairach). Sub-regions of the ventral PFC were identified as described by Ongur et al (2003).

Behavioral data were analyzed using the Statistical Package for the Social Sciences (SPSS 16, SPSS Inc., Chicago). Acquisition of reward contingencies was assessed using a one-sample *t*-test against zero on the adaptive reward learning measures. Effects of block on adaptive and aberrant reward learning, omission errors, premature responses and earnings were assessed using repeated-measures analysis of variance, with block (one, two or three) and probability (high or low) as the within-subjects factor where appropriate; the Huynh-Feldt correction was employed where significant non-sphericity was detected.

## Results

### Behavior on the SAT

Probability rating data acquired at the end of each block suggested that participants were able to acquire the stimulus–reward associations effectively, though not perfectly. Probability ratings were significantly higher for high-probability reward cues relative to low-probability reward cues (*t*(18) = 6.7, *p* < 0.000001; mean explicit measure of adaptive reward learning: 31.2 mm (SD 20.2 mm)). The explicit measure of adaptive reward learning progressed significantly over the course of the task (probability × block interaction: *F*(1.6, 28.4) = 9.7, *p* < 0.005, ɛ = 0.79). The mean explicit measure of aberrant reward learning (i.e. the absolute difference in probability rating between the two levels of the irrelevant stimulus dimension) was 11.2 mm (SD 9.1 mm), but the explicit measure of aberrant reward learning did not change significantly over the course of the task (main effect of block: *F*(2, 36) = 1.6, *p* > 0.1).

RT data acquired during the task confirmed that participants were able to use the reward associations to guide their responding adaptively, as reported previously (Roiser et al., 2009). Following the exclusion of an outlier who scored 3.5 SDs lower than the rest of the group on the implicit adaptive reward learning measure, participants responded significantly more quickly on high- relative to low-probability trials (*t*(17) = 2.2, *p* = 0.046; mean implicit measure of adaptive reward learning: 6.5 ms (SD 12.7 ms)), though the implicit measure of adaptive reward learning did not change significantly over the course of the task (probability × block interaction: *F* < 1). Mean implicit aberrant reward learning (i.e. the absolute difference in RT between the two levels of the irrelevant stimulus dimension) was 15.7 ms (SD 8.5 ms), and also did not change significantly over the course of the task (main effect of block: *F*(2, 36) = 1.8, *p* > 0.1). Participants responded with similar speed on trial-types subsequently rated as ‘high-probability' reward on the task-irrelevant dimension (mean 292.8 ms (SD 24.6 ms)) relative to those subsequently rated as ‘low-probability' reward (mean 293.6 ms (SD 21.0 ms)) (*t*(18) < 1).

The explicit measure of adaptive reward learning correlated significantly with the implicit measure of adaptive reward learning (*r* = 0.58, *p* = 0.010), though the implicit and explicit aberrant reward learning measures were uncorrelated (*r* = − 0.145, *p* = 0.55). Adaptive and aberrant reward learning measures were uncorrelated, both for the implicit (*r* = − 0.130, *p* = 0.61) and explicit (*r* = 0.333, *p* = 0.18) measures.

The amount of money won per rewarded trial was significantly higher on high-probability reward trials (*N* = 28 per block, mean £0.22 (SD £0.05) per reward) relative to low-probability reward trials (*N* = 4 per block, mean £0.18 (SD £0.07) per reward) (*t*(18) = 2.2, *p* = 0.042), again suggesting that the cue–reward associations influenced participants' responding. However, participants won similar amounts of money per trial on trial-types subsequently rated as ‘high-probability' reward on the task-irrelevant stimulus dimension (*N* = 16 per block, mean £0.22 (SD £0.06) per reward) relative to those subsequently rated as ‘low-probability' reward (*N* = 16 per block, mean £0.21 (SD £0.05) per reward) (*t*(18) = 1.1, *p* = 0.3). Overall, participants won an average of £6.76 (SD £0.36) per block, with more money won on later blocks (main effect of block: *F*(2,36) = 11.2, *p* < 0.001), again suggestive of learning over the course of the task. Notably, participants' average response time did not vary significantly over blocks (*F*(2,36) = 2.2, *p* = 0.13), suggesting that this increase in earnings was not simply related to an overall speeding of responses on later blocks. Across participants, the explicit measure of adaptive reward learning showed a trend toward correlating with the total amount money won (*r* = 0.41, *p* = 0.08). However, neither IQ not digit span correlated significantly with any measure of reward learning (either implicit or explicit measures: *r* < 0.3, *p* > 0.25 in all cases).

Participants made an average of 2.7 (SD 2.0) premature responses per block and 4.5 (SD 5.2) omissions per block, with no difference between high- and low-probability rewarded trials and no main effect of block (*p* > 0.1 for all).

### fMRI data

#### Parametric modulation of reinforcement outcome

As expected, parametric modulation of reward outcome yielded robust hemodynamic responses in the ventral striatum, which survived whole-brain FWE correction at both the voxel-level (*p* = 0.0001 (WBC)) and the cluster-level (*p* = 5 × 10^− 16^ (WBC)), suggesting that increasing monetary gain robustly engaged the brain's reward system in a parametric fashion. Parametric modulation of reward outcome also yielded responses in a number of ventral PFC structures, including pre-genual cingulate, lateral PFC and lateral orbitofrontal cortex, as well as bilateral inferior temporal gyrus, left precentral gyrus and right supra-marginal gyrus, all of which survived whole-brain FWE correction at the cluster-level (see Table S1 for full list of coordinates).

#### Adaptive reward prediction

**Fig. 2:**
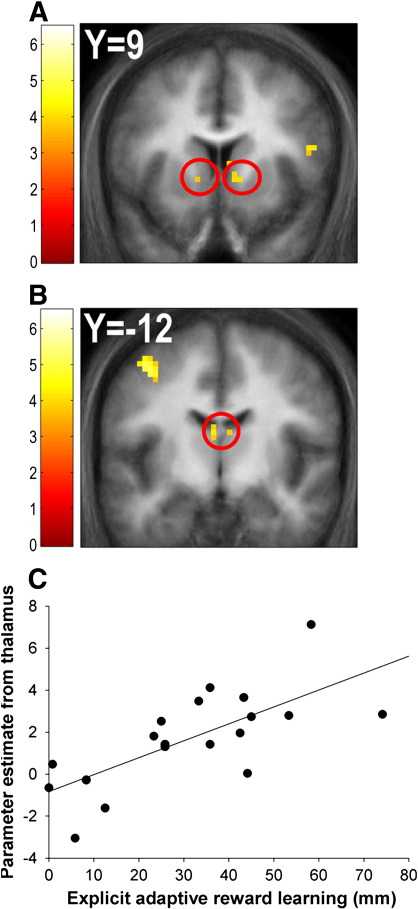
Hemodynamic responses associated with high- relative to low-probability reward cues. Presenting cues associated with a high relative to low probability of reinforcement elicited responses bilaterally in the ventral striatum (peak coordinates: right [*x* = 12, *y* = 12, *z* = − 3]; left [*x* = − 12, *y* = 9, *z* = − 3]) (A) and medial dorsal thalamus (peak coordinates: right [*x* = 3, *y* = − 9, *z* = 9]; left [*x* = − 3, *y* = − 9, *z* = 9]) (B). Responses in the thalamus were strongly correlated with the degree of explicit adaptive reward learning across participants (peak coordinates: right [*x* = 6, *y* = − 12, *z* = 15]; left [*x* = − 3, *y* = − 12, *z* = 15], *r* = 0.71 (plotted in scatterplot)) (C).

Contrasting the effect of presenting cue features associated with a high relative to low probability of reward revealed a network of structures, all of which survived whole-brain FWE correction at the cluster-level, including a large cluster in the midbrain (the peak voxel of which corresponded to the ventral tegmental area: VTA) extending to bilateral MD thalamus (*p* = 0.0004 (WBC)), bilateral superior temporal gyrus (right: *p* = 0.00003 (WBC); left: *p* = 0.000001 (WBC)), posterior insula (*p* = 0.021 (WBC)) and cerebellum (*p* = 0.025 (WBC)), as well as bilateral ventral striatum, which survived SVC FWE correction at the voxel-level (right: [*x* = 12, *y* = 12, *z* = − 3], *p* = 0.023 (SVC); left [*x* = − 12, *y* = 9, *z* = − 3], *p* = 0.045 (SVC)) (Fig. 2A; see Table S2 for a full list of coordinates).

**Fig. 3:**
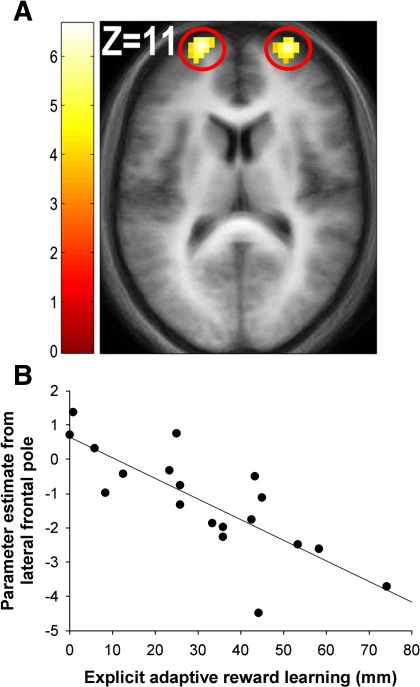
Hemodynamic responses associated with low- relative to high-probability reward cues. (A) Presenting cues associated with a low relative to high probability of reinforcement elicited responses in the lateral frontal pole bilaterally (peak coordinates: right [*x* = 24, *y* = 54, *z* = 6]; left [*x* = − 21, *y* = 63, *z* = 12])), which were strongly correlated with the degree of explicit adaptive reward learning across participants (peak coordinates: right [*x* = 9, *y* = 69, *z* = 6]; left [*x* = − 27, *y* = 48, *z* = 18], *r* = − 0.80 (plotted in scatterplot)) (B).

The reverse contrast revealed a network of PFC structures, all of which survived whole-brain FWE correction at the cluster-level, including DLPFC (*p* = 0.006 (WBC)) and lateral frontal pole bilaterally (right: *p* = 0.00005 (WBC); left: *p* = 0.001 (WBC)) (Fig. 3; see Table S2 for a full list of coordinates).

Including the explicit adaptive reward learning measure as a covariate in this analysis allowed the identification of regions where inter-individual differences in hemodynamic responses associated with adaptive reward prediction were correlated with inter-individual variation in explicit adaptive reward learning measures. In other words, we could identify regions whose differential activation to high- relative to low-probability cue features correlated with our explicit measure of adaptive reward learning across participants. This analysis revealed correlations surviving whole-brain FWE correction at the cluster-level in the dorsal anterior cingulate (*p* = 0.0002 (WBC)) and precentral gyrus (*p* = 0.027 (WBC)), as well as bilateral MD thalamus, which survived SVC FWE correction at the voxel-level (right: [*x* = − 3, *y* = − 12, *z* = 15], *p* = 0.021 (SVC); left [*x* = − 3, *y* = − 9, *z* = 12], *p* = 0.026 (SVC)) (Figs. 2B and C; see Table S2 for a full list of coordinates). Including the implicit adaptive reward learning measure as a covariate also revealed a correlation in the right MD thalamus that survived SVC FWE correction at the voxel-level ([*x* = 3, *y* = − 15, *z* = 6], *p* = 0.044 (SVC); see Table S2 for a full list of coordinates).

The reverse contrast, i.e. testing for negative correlations between hemodynamic responses associated with adaptive reward prediction and explicit measures of adaptive reward learning across participants, revealed correlations surviving whole-brain FWE correction at the cluster-level in the occipital cortex (*p* = 0.004 (WBC)) and left lateral frontal pole (overlapping with the frontopolar main effect identified above: *p* = 0.00009 (WBC)), as well a correlation that showed a trend toward whole-brain FWE corrected significance in the right frontal pole (*p* = 0.097 (WBC)) (Fig. 3B; see Table S2 for a full list of coordinates). Including the implicit adaptive reward learning measure as a covariate also revealed a correlation in the left lateral frontal pole that survived SVC FWE at the voxel-level (SVC based on a mask of the orthogonal reverse main effect at *p* < 0.001 (uncorrected): [*x* = − 24, *y* = 54, *z* = 18], *p* = 0.004 (SVC); see Table S2 for a full list of coordinates).

#### Aberrant reward prediction

The contrast between subjective ‘high’- relative to subjective ‘low-probability' cue features (defined according to the direction of each participant's block-specific explicit measure of aberrant reward learning across the task-irrelevant stimulus dimension—see Materials and methods) did not reveal any effects that survived whole-brain FWE correction, either at the cluster-level or the voxel-level.

**Fig. 4:**
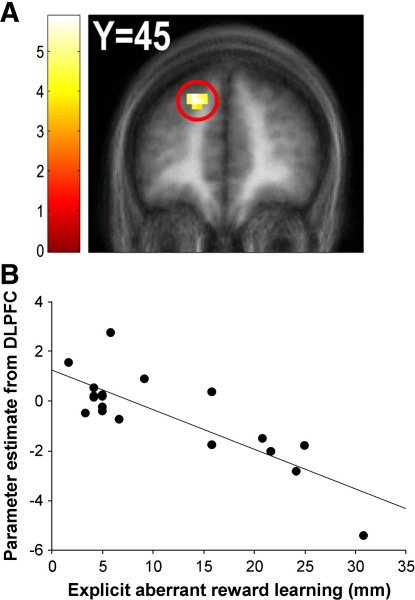
Hemodynamic responses associated with explicit aberrant reward learning. Differential response to two cues associated with equal (50%) probability of reward in the dorsolateral prefrontal cortex (peak coordinates: [*x* = − 18, *y* = 45, *z* = 36]) (A) was strongly correlated with the degree of explicit aberrant reward learning pertaining to those stimuli across participants (*r* = − 0.82) (B).

However, our statistical model also included the explicit measure of aberrant reward learning measure as a covariate; this allowed the identification of regions where inter-individual differences in hemodynamic responses associated with aberrant reward prediction were correlated with inter-individual variation in the explicit measure of aberrant reward learning. In other words, we could identify regions whose differential activation to cue features at the two levels of the irrelevant stimulus dimension, both associated with 50% probability of reward, correlated with our explicit measure of aberrant reward learning. This analysis revealed two regions that survived whole-brain FWE correction at the cluster-level, one in the right middle temporal gyrus (MTG) where responses were positively correlated with aberrant reward learning (*p* = 0.002 (WBC)), the other in the left DLPFC where responses were negatively correlated with aberrant reward learning (*p* = 0.043 (WBC)) (Fig. 4; see Table S3 for a full list of coordinates). There were no regions where hemodynamic responses associated with aberrant reward prediction were significantly correlated with inter-individual variation in implicit aberrant reward learning measures, even at an uncorrected threshold of *p* < 0.001, 10 contiguous voxels.

## Discussion

A major finding of this study was that variation across participants in aberrant reward learning pertaining to cues associated with identical reward probabilities was strongly associated with differential DLPFC and MTG responses to those stimuli. We also found, consistent with previous studies (Knutson et al., 2001), that presenting cues with a strong reward association elicited hemodynamic responses in the VTA, MD thalamus and ventral striatum, and that responses in the MD thalamus correlated with inter-individual variability in the explicit measure of adaptive reward learning. Finally, we demonstrate that simply presenting cues with a low relative to high probability of reward elicited robust hemodynamic responses in the lateral frontal pole and DLPFC, and that responses in the lateral frontal pole correlated negatively with inter-individual differences in the extent of explicit adaptive reward learning.

Presenting cues associated with a high probability of reward relative to those with a low probability of reward elicited hemodynamic responses in the midbrain (corresponding to the VTA), MD thalamus and ventral striatum (Fig. 2A). Furthermore, the magnitude of hemodynamic responses in the MD thalamus was strongly correlated with the degree to which participants learned to distinguish the reward probabilities of the high- and low-probability cues (Figs. 2B and C). Since we did not record arterial pulsation during the scan we cannot exclude the possibility that the hemodynamic signal change in the midbrain may have been confounded by pulsation of brain stem arteries. Nevertheless, the location of the peak voxel identified in the midbrain corresponded to the anatomical location of the VTA (Mai et al., 2003), and the increased BOLD signal in the ventral striatum and MD thalamus, both innervated by dopamine, is consistent with an explanation in terms of increased input from the VTA.

The MD thalamus, which receives afferent inputs from the ventral striatum both directly and indirectly via the ventral pallidum, sends efferent projections to the ventral part of the prefrontal cortex (Alexander et al., 1986; Lawrence et al., 1998). The ventral prefrontal cortex itself projects back to the ventral striatum, thus closing a circuit involved in the automatic processing of emotionally relevant environmental stimuli, the ‘affective’ cortico–striatal–thalamic loop (Ongur and Price, 2000). Therefore, we suggest that the responses we identified reflect the arousing and invigorating effect of stimuli associated with reward, probably mediated by dopamine release in the ventral striatum (Berridge and Robinson, 1998). We further speculate that the MD region of the thalamus plays a role in orienting attention toward motivationally salient stimuli, as demonstrated in previous studies (Small et al., 2005), in a similar fashion to that demonstrated for the pulvinar nucleus of the thalamus in mediating attention toward visually salient stimuli (Robinson and Petersen, 1992; Morris et al., 1997).

Presenting cues with a low probability of reward relative to those with a high probability of reward elicited strong responses in the lateral frontal pole (Fig. 3A) and DLPFC. Furthermore, the magnitude of hemodynamic response in the lateral frontal pole was strongly correlated with the degree to which participants learned to distinguish the reward associations of the high-probability and low-probability cues (Fig. 3B). The involvement of DLPFC is consistent with previous findings implicating this region in associative learning (Corlett et al., 2004). Responses in the more ventral lateral frontal pole area have been reported during the processing of internal representations as opposed to external stimuli, or ‘mind-wandering’ (Christoff et al., 2004; Burgess et al., 2007). Furthermore, as we also found, such responses tend to occur under conditions where participants respond more slowly to stimuli (see Gilbert et al., 2006 for a meta-analysis). Responses in this region have also been reported during instrumental reinforcement learning when participants chose a low-probability reward stimulus over a high-probability reward stimulus (the ‘exploratory choice’ condition in Daw et al., 2006). However, in the present study, simply presenting low-probability reward cues was sufficient to elicit responses in the lateral frontal pole.

By contrast, the presentation of cue features associated with identical reward probabilities (i.e. comparing the two levels of the task-irrelevant stimulus dimension) did not elicit consistent differential responses across participants. This is perhaps unsurprising, as on average participants exhibited very little aberrant reward learning. However, our covariate analysis revealed robust relationships between the *degree* of aberrant reward learning across participants and differential responses to irrelevant cue features in the DLPFC and MTG. Irrelevant cue features that were erroneously inferred to be associated with a higher probability of reward elicited smaller DLPFC responses and greater MTG responses, and these differential regional responses were expressed more strongly the higher participants scored on the explicit aberrant reward learning measure. One possibility is that the responses in these regions simply reflect the erroneous prediction of lower vs higher reward, given that the same DLPFC region was evident in the adaptive reward learning contrast (see above). However, this cannot be the complete explanation because there was a spatial dissociation between fictitious (aberrant) and veridical (adaptive) reward-related responses. Aberrant reward prediction signals were not evident in sub-cortical regions such as striatum, thalamus and midbrain, which were apparent in adaptive reward learning analysis, whereas the converse was true for the MTG. This suggests that the processing of fictitious and veridical value is qualitatively different and engages distinct (if overlapping) systems. We now consider the nature of this difference.

Our results speak to an ongoing discussion in the literature regarding which aspects of stimuli drive associative learning. On the one hand, Mackintosh (1975) suggested that the associability of a stimulus is determined by how *reliably* it predicts an outcome. Thus highly predictive (i.e. low uncertainty) cues should be most associable, and over time non-predictive cues are learned to be ignored; this type of mechanism would encourage adaptive reward learning on the SAT. On the other hand, Pearce and Hall (1980) proposed that cues with *uncertain* consequences are most associable, more strongly capturing attention; such a mechanism could possibly contribute to aberrant reward learning on the SAT.

One way to express uncertainty about possible outcomes is the information theoretic concept of entropy, which represents the average surprise over all possible outcomes, in our case either presence or absence of reward (e.g. see Strange et al., 2005). On the SAT, the uncertainty about the outcome is equal for both ends of the task-relevant stimulus dimension: reward can be either present with 87.5% probability and absent with 12.5%, or vice versa, both corresponding to an entropy of 0.377. Contrasting these cue features revealed robust responses in a well-characterized circuit innervated by dopaminergic projections as described above, suggesting an important role for the affective cortico-striatal loop in associative learning from predictable cues (Mackintosh, 1975).

On the other hand, the two ends of the task-irrelevant stimulus dimension on the SAT are both associated with a highly uncertain indication of reward outcome (50% probability, corresponding to a maximal entropy of 0.693). According to the Pearce and Hall (1980) model, these cue features should be highly associable. When probing aberrant reward learning by comparing the two levels of the task-irrelevant stimulus dimension, we did not find any significant responses across the group as a whole; however, we did observe significant correlations between the degree of aberrant reward learning and responses to irrelevant cues in DLPFC and MTG. These regions have both previously been reported to be sensitive to changes in uncertainty (represented by entropy: Bischoff-Grethe et al., 2000). Therefore, the correlations we observed across participants between responses in these regions and the degree of aberrant reward learning might reflect individual preferences in how uncertain cues are evaluated (see also Huettel et al., 2006; Chew et al., 2008).

It should be noted that this somewhat speculative interpretation also implies that the responses we observed in the adaptive reward prediction contrast should reflect associative learning under low uncertainty. Some of these regions, however, were previously reported to correlate positively with the uncertainty of reward outcome (Fiorillo et al., 2003; Preuschoff et al., 2006). This apparent contradiction may be related to the fact that these previous studies assessed responses during a delay period immediately preceding reward delivery, while we modeled responses to the cue stimulus itself. Perhaps even more importantly, in these previous studies the probability distribution of outcomes was known to the subjects (due to overtraining or instruction, respectively) whereas in our study it was not (c.f. the distinction between “risk” and “ambiguity” in the economics literature: Camerer and Weber, 1992; Huettel et al., 2006).

In summary, we demonstrate that the extent of aberrant reward learning across individuals is strongly associated with the magnitude of differential MTG and DLPFC responses to cues erroneously inferred to differ in terms of reward association. By contrast, adaptive reward prediction responses were identified in a network of structures including regions of the thalamus, striatum and prefrontal cortex comprising the ‘affective’ cortico-striatal loop. Following this initial study in healthy volunteers, it will be important in future work to assess the neural mechanisms underpinning aberrant reward processing in patients with psychosis, since maladaptive reinforcement signaling has been posited as a central mechanism underlying psychotic symptoms (Kapur, 2003; Jensen et al., 2008; Murray et al., 2008). In particular, it will be of interest to test whether DLPFC and MTG responses to irrelevant cues during aberrant reward learning correlate with the severity of psychotic symptoms.

## Declaration of interest

The authors declare that they have no relevant conflicts of interest.
