# Supplementary material for: Adaptive and aberrant reward prediction signals in the human brain
Source: Neuroimage. 2010 Apr 1;50(2):657–64. doi: 10.1016/j.neuroimage.2009.11.075 (PMC2828543; doi:10.1016/j.neuroimage.2009.11.075)
Supplement: Supplementary file 1 — Supplementary Material [file mmc1.doc]

**Table S1 – Regions identified by parametric modulation of reward** outcome.

| **Region** | **Right/left** | **MNI co-ordinates** | | | **Cluster size^1^** | **Z value** |
| --- | --- | --- | --- | --- | --- | --- |
|  |  | **X** | **Y** | **Z** |  |  |
| Ventral striatum  Ventral striatum | R | 15 | 18 | -6 | 589^†^ | 5.89* |
|  | L | -9 | 12 | 3 |  | 4.81* |
| Inferior temporal gyrus | L | -54 | -54 | -18 | 38^†^ | 5.08* |
| Inferior temporal gyrus | R | 57 | -48 | -18 | 24 | 4.80* |
| Pre-genual cingulate | M | 3 | 45 | 12 | 312^†^ | 4.49 |
| Lateral prefrontal cortex | R | 54 | 36 | 24 | 84^†^ | 4.46 |
| Supra-marginal gyrus | L | -51 | -42 | 48 | 18 | 4.29 |
| Lateral orbitofrontal cortex | R | 45 | 48 | -12 | 56^†^ | 4.26 |
| Cerebellum | L | -6 | -81 | -33 | 18 | 4.25 |
| Insula | R | 48 | 9 | 33 | 35 | 4.08 |
| Pre-central gyrus | L | -45 | 3 | 33 | 38^†^ | 3.99 |
| Supra-marginal gyrus | R | 48 | -51 | 54 | 53^†^ | 3.95 |
| Superior temporal gyrus | R | 66 | -36 | 0 | 24 | 3.72 |
| Supra-marginal gyrus | R | 54 | -36 | 57 | 11 | 3.62 |
| Medial temporal gyrus | R | 60 | -33 | -9 | 10 | 3.52 |
| Ventrolateral prefrontal cortex | L | -48 | 42 | 12 | 12 | 3.36 |

Coordinates correspond to MNI space and denote the distance in mm from the anterior commissure, with positive x = right of midline, positive y = anterior to the anterior commissure, and positive z = dorsal to a plane containing both the anterior and the posterior commissures.

Abbreviations: L – left; M – midline; R – right; ^1^ Corresponds to a threshold of p<0.001 (uncorrected), minimum cluster-size 10 voxels. * Survives whole-brain family-wise error correction for multiple comparisons at the voxel-level; ^†^ Survives whole-brain family-wise error correction for multiple comparisons at the cluster-level.

**Table S2 – Regions identified by the contrast of high- relative to low-probability CS presentation (adaptive reward prediction)**.

|  | **Right/left** | **MNI co-ordinates** | | | **Cluster size^1^** | **Z value** |
| --- | --- | --- | --- | --- | --- | --- |
| **High minus low probability** |  | **X** | **Y** | **Z** |  |  |
| Superior temporal gyrus | L | -63 | -24 | 12 | 209^†^ | 4.60 |
| Midbrain (VTA)  Medial dorsal thalamus  Medial dorsal thalamus | L | -9 | -27 | -9 | 108^†^ | 4.46 |
|  | R | 3 | -9 | 9 |  | 3.55 |
|  | L | -3 | -9 | 9 |  | 3.71 |
| Posterior insula | L | -39 | -18 | -9 | 52^†^ | 4.42 |
| Superior temporal gyrus | R | 66 | -30 | 12 | 151^†^ | 4.01 |
| Cerebellum | R | 6 | -63 | -15 | 50^†^ | 3.75 |
| Ventral striatum | R | 12 | 12 | -3 | 6 | 3.35^#^ |
| Ventral striatum | L | -12 | 9 | -3 | 1 | 3.10^#^ |
| **Low minus high probability** |  |  |  |  |  |  |
| Polar prefrontal cortex | L | -21 | 63 | 12 | 90^†^ | 4.66 |
| Polar prefrontal cortex | R | 24 | 54 | 6 | 139^†^ | 4.51 |
| Inferior temporal gyrus | R | 36 | 15 | -36 | 23 | 4.48 |
| DLPFC (superior frontal gyrus) | L | -24 | 36 | 45 | 69^†^ | 3.83 |
| Posterior cingulate cortex | R | 12 | -48 | 36 | 30 | 3.71 |
| Supra-marginal gyrus | R | 48 | -60 | 36 | 37 | 3.47 |
| **Positive covariation with adaptive learning (explicit)** |  |  |  |  |  |  |
| Dorsal anterior cingulate | M | 0 | 9 | 48 | 113^†^ | 4.55 |
| Premotor cortex | M | -3 | 3 | 69 | 10 | 4.11 |
| Precentral gyrus | L | -39 | -12 | 51 | 47^†^ | 3.99 |
| Cerebellum | M | 0 | -30 | -39 | 15 | 3.87 |
| Motor cortex | R | 39 | -6 | 51 | 29 | 3.85 |
| Superior parietal lobe | R | 33 | -48 | 45 | 10 | 3.73 |
| Premotor cortex | R | 15 | -3 | 63 | 12 | 3.55 |
| Medial dorsal thalamus | R | 6 | -12 | 15 | 4 | 3.14^#^ |
| Medial dorsal thalamus | L | -3 | -12 | 15 | 4 | 3.41^#^ |
| **Positive covariation with adaptive learning (implicit)** |  |  |  |  |  |  |
| Occipital cortex | R | 21 | -90 | 9 | 16 | 4.46 |
| Occipital cortex | R | 3 | -72 | 18 | 36 | 4.02 |
| Medial dorsal thalamus | R | 3 | -15 | 6 | 1 | 3.12^#^ |
| **Negative covariation with adaptive learning (explicit)** |  |  |  |  |  |  |
| Occipital cortex | L | -30 | -66 | -3 | 71^†^ | 4.72 |
| Polar prefrontal cortex | R | 9 | 69 | 6 | 33 | 4.39 |
| Inferior temporal gyrus | R | 45 | 6 | -42 | 20 | 4.09 |
| Polar prefrontal cortex | L | -27 | 48 | 18 | 124^†^ | 4.08 |
| Occipital cortex | R | 21 | -90 | 24 | 31 | 4.03 |
| Cerebellum | L | -30 | -75 | -42 | 20 | 3.74 |
| Precuneus | M | 3 | -60 | 27 | 11 | 3.60 |
| Parahippocampal gyrus | L | -27 | -39 | -18 | 10 | 3.60 |
| **Negative covariation with adaptive learning (implicit)** |  |  |  |  |  |  |
| Polar prefrontal cortex | L | -24 | 54 | 18 | 31 | 4.33^#^ |
| Parahippocampal gyrus | L | -18 | -39 | -9 | 23 | 4.08 |
| Fusiform gyrus | L | -33 | -54 | -3 | 10 | 4.07 |
| Middle temporal gyrus | R | 57 | 3 | -21 | 23 | 3.82 |
| Fusiform gyrus | R | 33 | -42 | -12 | 33 | 3.8 |
| Parietal/occipital transition zone | L | -36 | -84 | 33 | 17 | 3.76 |
| Parietal/occipital transition zone | R | 33 | -81 | 42 | 33 | 3.7 |

Coordinates correspond to MNI space and denote the distance in mm from the anterior commissure, with positive x = right of midline, positive y = anterior to the anterior commissure, and positive z = dorsal to a plane containing both the anterior and the posterior commissures.

Abbreviations: L – left; M – midline; R – right; VTA – ventral tegmental area; DLPFC – dorsolateral prefrontal cortex; ^1^ Corresponds to a threshold of p<0.001 (uncorrected), minimum cluster-size 10 voxels. ^†^ Survives whole-brain family-wise error correction for multiple comparisons at the cluster-level. ^#^ Survives small-volume adjusted family-wise error correction for multiple comparisons at the voxel-level.

**Table S3 – regions identified by the contrast of subjective ‘high’- relative to subjective ‘low’-probability CS presentation (aberrant reward prediction)**.

|  | **Right/left** | **MNI co-ordinates** | | | **Cluster size^1^** | **Z value** |
| --- | --- | --- | --- | --- | --- | --- |
| **Subjective ‘high’ minus ‘low’ probability** |  | **X** | **Y** | **Z** |  |  |
| Superior temporal gyrus | R | 54 | -42 | 24 | 14 | 4.25 |
| Post-central gyrus | R | 54 | -24 | 24 | 14 | 3.90 |
| **Subjective ‘low’ minus ‘high’ probability** |  |  |  |  |  |  |
| No clusters survive threshold |  |  |  |  |  |  |
| **Positive covariation with aberrant learning (explicit)** |  |  |  |  |  |  |
| Primary visual cortex | R | 18 | -84 | 6 | 18 | 4.42 |
| Cerebellum | R | 18 | -54 | -33 | 23 | 4.11 |
| Ventrolateral prefrontal cortex | R | 24 | 36 | 12 | 18 | 4.08 |
| Inferior temporal gyrus | R | 39 | -54 | -21 | 24 | 3.91 |
| Cerebellum | M | 3 | -63 | -39 | 21 | 3.80 |
| Middle temporal gyrus | R | 48 | -66 | 12 | 67^†^ | 3.78 |
| Orbitofrontal cortex | R | 21 | 27 | 0 | 16 | 3.63 |
| Ventrolateral prefrontal cortex | L | -21 | 36 | 9 | 10 | 3.62 |
| **Positive covariation with aberrant learning (implicit)** |  |  |  |  |  |  |
| No clusters survive threshold |  |  |  |  |  |  |
| **Negative covariation with aberrant learning (explicit)** |  |  |  |  |  |  |
| DLPFC (superior frontal gyrus) | L | -18 | 45 | 36 | 35^†^ | 4.28 |
| DLPFC (middle frontal gyrus)^2^ | L | -33 | 21 | 51 | 16 | 3.92 |
| Fusiform gyrus | L | -33 | -60 | -6 | 12 | 3.74 |
| **Negative covariation with aberrant learning (implicit)** |  |  |  |  |  |  |
| No clusters survive threshold |  |  |  |  |  |  |

Coordinates correspond to MNI space and denote the distance in mm from the anterior commissure, with positive x = right of midline, positive y = anterior to the anterior commissure, and positive z = dorsal to a plane containing both the anterior and the posterior commissures.

Abbreviations: L – left; M – midline; R – right; DLPFC – dorsolateral prefrontal cortex. ^1^ Corresponds to a threshold of p<0.001 (uncorrected), minimum cluster-size 10 voxels. ^2^ A similar effect was also detected in the right DLPFC [x=45, y=27, z=33], but the cluster did not survive the extent threshold. ^†^ Survives whole-brain family-wise error correction for multiple comparisons at the cluster-level.
